# Retrograde embolization technique of the right gastric artery during the implantation of port-catheter system for hepatic arterial infusion chemotherapy

**DOI:** 10.1016/j.jimed.2020.10.004

**Published:** 2020-10-12

**Authors:** Jungang Hu, Guang Cao, Liang Xu, Kanglian Zheng, Xu Zhu, Renjie Yang, Xiao Wang, Xiaodong Wang

**Affiliations:** a Department of Radiology, Civil Aviation General Hospital, Beijing, 100123, China; b Department of Interventional Radiology, Peking University Cancer Hospital & Institute, Key Laboratory of Carcinogenesis and Translational Research(Ministry of Education), Beijing, 100142, China; c Department of Epidemiology and Biostatistics, Peking University Sixth Hospital, Beijing, 100191, China

**Keywords:** Hepatic arterial infusion chemotherapy, Right gastric artery embolization, Left gastric artery, Port-catheter system, Acute gastroduodenal mucosal toxicity

## Abstract

**Objective:**

This study aimed to introduce and evaluate a new embolization technique for the right gastric artery (RGA) during percutaneous implantation of a port-catheter system for hepatic arterial infusion chemotherapy (HAIC).

**Methods:**

From January 2013 to January 2017**,** 159 patients with unresectable advanced liver cancer underwent percutaneous implantation of a port-catheter system. In 86 of these patients (56 men; aged 28–88 years; mean: 60.6 ​± ​12.0 years), in whom the RGA was obvious on arteriography, embolization of RGA was attempted using microcoils to protect the gastric mucosa during HAIC. In the first phase (first three years), antegrade embolization of the RGA using a 2.7 Fr microcatheter was performed in 55 patients. In the second phase (next two years), embolization of the RGA was attempted by combining antegrade embolization and retrograde embolization through the left gastric artery (LGA) in 31 patients. The success rates and the incidence of acute gastroduodenal mucosal toxicity (AGMT) in these two groups were compared.

**Results:**

The total success rate of the RGA embolization was 70.9%. The success rate was 83.9% in 31 patients who underwent combined antegrade and retrograde embolization, which was significantly higher than that of antegrade embolization alone (63.6%) performed in 55 patients (*p* ​= ​0.047). No complications related to embolization of RGA were documented. The incidence of AGMT was 29.1% (16/55) in patients in the first phase, which was significantly higher than that in the patients in the second phase (9.7%, 3/31) (*p* ​= ​0.037).

**Conclusion:**

A combination of retrograde embolization via LGA could increase the success rates of RGA embolization and reduce the incidence of AGMT after HAIC.

## Introduction

As an arterial directed interventional treatment, hepatic artery infusion chemotherapy (HAIC) has been gradually used and was proved to be effective in hepatocellular carcinoma, cholangiocarcinoma, and colorectal liver metastasis.^1^, ^2^, ^3^ To facilitate long-term administration of anticancer agents, a permanent arterial port-catheter system has been implanted to allow repetitive HAIC.^3^ Recent advances in minimally invasive techniques allow percutaneous placement of catheter-port systems for HAIC.^4^ However, the major complication of HAIC is acute gastroduodenal mucosal toxicity (AGMT) manifesting as antral gastritis, gastric ulcer, duodenitis, and duodenal ulcer, which results from the chemical irritation caused by the infusion of chemotherapeutic agents into the gastroduodenum through arteries originating from the common hepatic artery.^5^, ^6^, ^7^ To prevent this complication, selective embolization of the arteries such as the gastroduodenal artery (GDA) supplying the adjacent organs has been suggested.^8^ However, even in patients with sufficient embolization of the GDA, gastromucosal lesions occurred in 3.2–47.5% of patients in whom the chemotherapeutic agents were distributed to the stomach wall via the preserved right gastric artery (RGA).^9^ Therefore some scholars strongly recommended the occlusion of the RGA additionally.^10^, ^11^, ^12^ HAIC with an implanted port-catheter system has been a widely used technique performed in a large number of cases in our institution; embolization of the RGA was always attempted for every patient in cases where the RGA was obviously visible on arterial angiogram. Because of the special anatomical features of the RGA, often a large angle bending back from the hepatic artery, antegrade catheterization is extremely difficult in some cases. With the left gastric artery (LGA) and RGA connection at the lesser curvature of the stomach, we attempted retrograde catheterization and embolization of the RGA from the LGA. This study examined the success rates of RGA embolization and compared the incidence of AGMT after performing a combination of retrograde and antegrade embolization with that after performing antegrade embolization alone.

## Material and methods

### Ethical approval

A waiver of authorization was obtained from the local ethics committee for this retrospective study. All clinical practices and observations were conducted in accordance with the Declaration of Helsinki. Informed consent was obtained from each patient before the study was conducted.

## Subjects

From January 2013 to January 2017, the data of 159 patients who were selected for HAIC with port-catheter system in our institution were retrospectively analyzed. Embolization of the RGA to prevent infusion of chemotherapeutic agents into the stomach was attempted in 86 of the 159 patients. Patient eligibility criteria for RGA embolization for HAIC were as follows: RGA was clearly evident on celiac or common hepatic arteriography. Very minute RGAs were excluded from embolization. Of the 86 patients, 29 patients had perihilar cholangiocarcinoma, 20 had intrahepatic cholangiocarcinoma, 7 had primary liver cancer (hepatocellular carcinoma, 6; mixed type, 1), 30 had metastatic liver cancer originating from colorectal cancer (*n* ​= ​22), gastric cancer (*n* ​= ​2), and gallbladder cancer (*n* ​= ​6). All patients were in the advanced unresectable stage, but with total or most tumors limited to the liver. Patient clinical characteristics are shown in Table 1.

**Table 1:** Clinical characteristics of patients underwent right gastric artery embolization.

Characteristics	Numbers
Sex	
Male	56 (65.1%)
Female	30 (34.9%)
Age (y)	60.6 ​± ​12.0
Cholangiocarcinoma	49 (57.0%)
Perihilar cholangiocarcinoma	29 (33.7%)
Intrahepatic cholangiocarcinoma	20 (23.3%)
Primary liver cancer	7 (8.1%)
Hepatocellular carcinoma	6 (7.0%)
Mixed type	1 (1.2%)
Metastatic liver cancer	30 (34.9%)
Colorectal cancer	22 (25.6%)
Gallbladder cancer	6 (7.0%)
Gastric cancer	2 (2.3%)

## Right gastric artery embolization

**Fig. 1:**
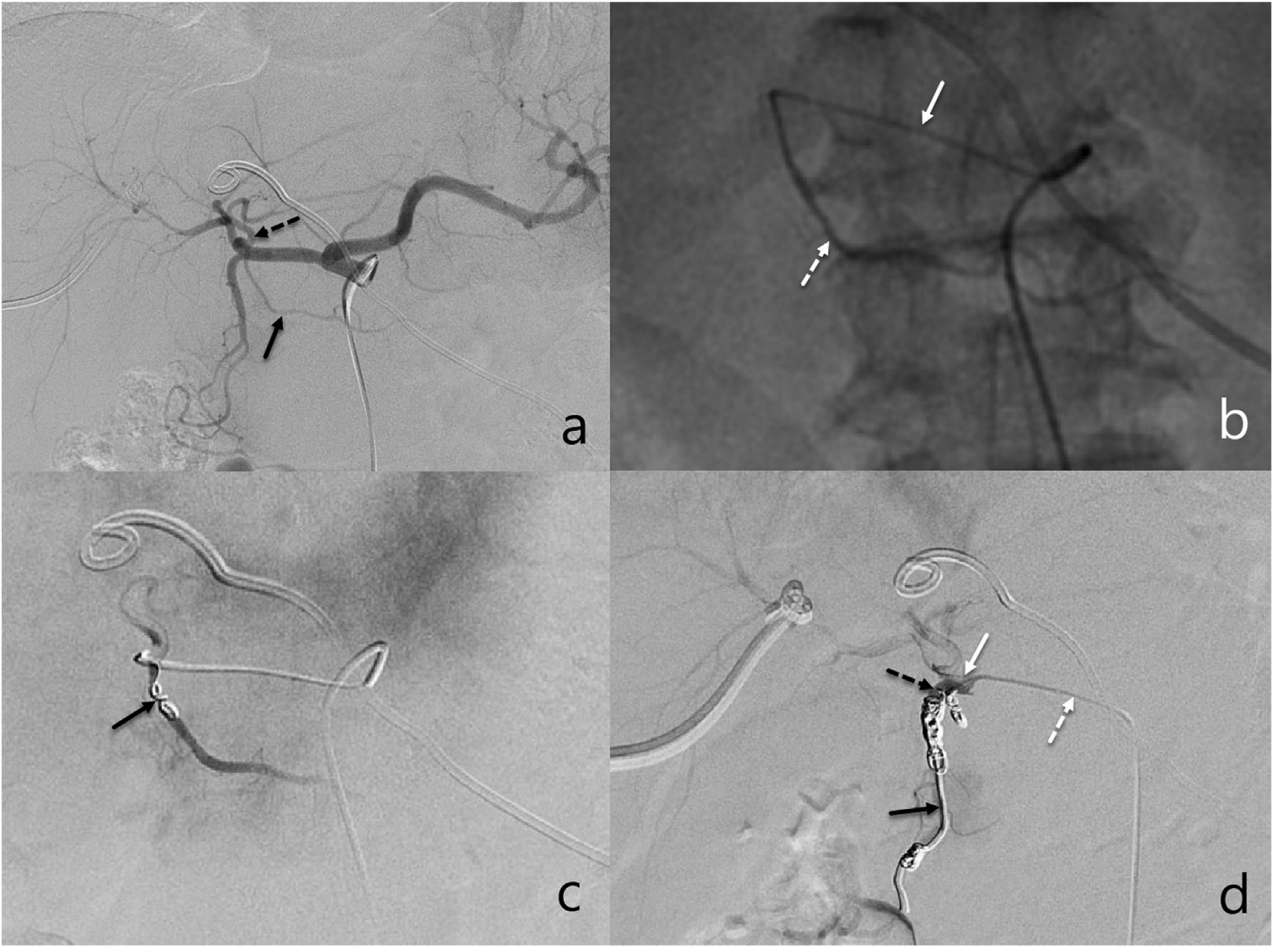
Antegrade embolization of RGA. 60-year-old man with perihilar cholangiocarcinoma. a. Celiac arteriogram obtained before implantation of port-catheter system showed the RGA (black arrow) arising from proximal the left hepatic artery (black dotted arrow). b. Right gastric arteriogram obtained through a coaxial microcatheter (white arrow) antegrade inserted into the RGA (white dotted arrow) showed its course. c. Left heptic arteriogram obtained after embolization of the proximal portion of the RGA with microcoils (black arrow) showed disappearance of blood flow into the RGA. d. Indwelling catheter tip (black arrow) was embolized in GDA (black dotted arrow) with the side hole (white arrow) at the proximal proper hepatic artery. Proper hepatic arteriogram obtained through side hole of port-catheter system (white dotted arrow) showed the whole hepatic artery tree, without blood flow into the RGA.

**Fig. 2:**
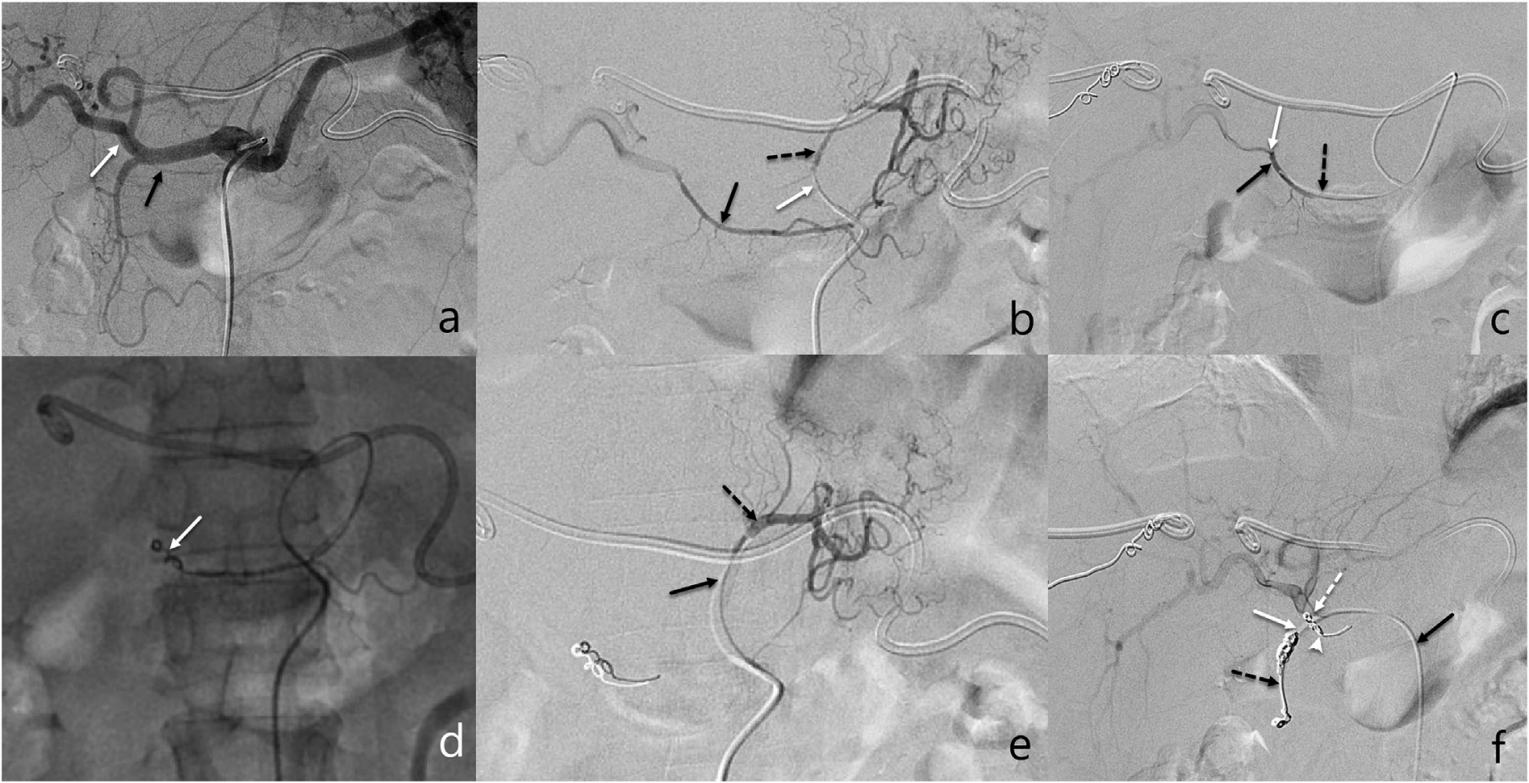
Retrograde embolization of RGA. 44-year-old man with perihilar cholangiocarcinoma. a. Celiac arteriogram obtained before implantation of port-catheter system showing the RGA (black arrow) arising from the proper hepatic artery (white arrow). b. Arteriogram obtained via microcatheter (micro catheter tip, white arrow) coaxially advanced 5-French catheter placed in LGA (black dotted arrow) showed the anastomotic branch (black arrow) between left and right gastric arteries. c. Microcatheter (black dotted arrow) was advanced retrograde across the anastomotic branch into proximal RGA, angiogram showed RGA (black arrow) arising site of distal proper hepatic artery (white arrow). d. Retrograde released microcoils (white arrow) in the RGA. e. Arteriogram obtained after embolization of RGA through microcatheter (black arrow) via LGA (black dotted arrow) confirmed completely embolized RGA. f. Indwelling catheter tip (black dotted arrow) was fixed in GDA (white arrow) using microcoils, with the side hole (white dotted arrow)at the proximal proper hepatic artery. Proper hepatic arteriogram through the side hole of port-catheter system (black arrow) showed proper hepatic artery and its branches, without appearance of the RGA (white arrow head).

RGA embolization was performed after hepatic arteriography using a 5 Fr Yashiro catheter (Terumo, Japan) before implantation of the port-catheter system. The origin and course of the RGA were determined by celiac or common hepatic arteriography. Once the origin of the RGA was identified, a 2.7 Fr microcatheter (Terumo, Japan) was antegradely advanced coaxially over a 5 Fr Yashiro catheter (Terumo, Japan) located in the common hepatic artery. Once antegrade RGA catheterization was achieved, embolization was performed using microcoils (Boston Scientific). In the first phase, i.e. the first three years of this study, only antegrade embolization was attempted. In the second phase, i.e. the last two years of this study, in the event that antegrade catheterization of the RGA failed, retrograde access to the RGA via the LGA was attempted (’‘retrograde route’‘). For retrograde catheterization of the RGA via the LGA, a 5 Fr catheter was placed in the origin of the LGA. Subsequently, a 2.7 Fr microcatheter (Terumo, Japan) was advanced coaxially into the LGA, and selective arteriography was performed to confirm the presence and morphology of the LGA–RGA anastomoses. The microcatheter was then retrogradely advanced from the LGA toward the origin of the RGA, where microcoils were deployed until complete occlusion of the RGA was ensured. Immediately after RGA embolization, interruption of the blood flow to the proximal portion of the RGA was confirmed by common hepatic or left gastric arteriography (see Fig. 1, Fig. 2).

## Port-catheter system implantation

Placement of the port-catheter system was subsequently performed in the same session. Catheter placement procedures were performed with the patient under local anesthesia. The indwelling catheter (Celsite 5F Implantofix 4,438,663 or Celsite 5F PSU ST305C 4,436,962, B. Braun Medical, France) was inserted from the right femoral artery. The port-catheter system was implanted using the fixed catheter tip technique.^4^ A side hole, through which the HAIC was administered, was created to open at the common hepatic artery just before the GDA arises, and the catheter tip was fixed to the GDA with microcoils (Cook, USA, or Boston Scientific, USA), and the GDA was embolized at the same time. The proximal end of the indwelling catheter was connected to a port implanted in the subcutaneous space. Details of the methods for port-catheter system placement are described elsewhere.^4^^,^^13^^,^^14^

## Hepatic arterial infusion chemotherapy

The most commonly used regimen for HAIC is oxaliplatin plus 5-fluorouracil (5-FU), which consists of infusion of oxaliplatin (35–40 ​mg/m^2^ for 2 ​h), followed by 5-FU (600–800 ​mg/m^2^ for 22 ​h) on days 1–3 every 3–4 weeks.^3^ Other HAIC regimens include irinotecan ​+ ​5-FU and gemcitabine ​+ ​nedaplatin. Maximally, 6 cycles of HAIC were used for patients without disease progression during treatment. Dose modifications were defined per protocol. Modifications and delays due to hematologic toxicity, abnormal liver and renal function, nausea, vomiting, and peripheral neuropathy were allowed.

## Port-catheter system follow-up

Before each infusion, patients underwent digital subtraction angiography (DSA) of the hepatic arterial circulation via injection of contrast (volume 6–8 ​mL, rate 1 ​mL/s) via the port system to confirm port patency, catheter tip, and presence or absence of recanalization of the RGA.

## Parameters investigated

Technical success rate and complications of RGA embolization with combined retrograde embolization and antegrade embolization alone and the incidence of AGMT after HAIC from the port-catheter system were investigated. The chi-square test was used for statistical comparison of these patients in the two phases. Results with *p* ​< ​0.05 were considered statistically significant. AGMT was diagnosed through the identification of gastroduodenal mucosal lesions using endoscopy or clinically based on persistent epigastric pain during or after hepatic arterial chemotherapy, which could be alleviated by a proton pump inhibitor with a gastric mucosal protective drug.^15^, ^16^, ^17^

## Results

Among the 86 study patients, the origins of the RGA were the proper hepatic artery (*n* ​= ​63, 73.3%), the common hepatic artery (*n* ​= ​10, 11.6%), the left hepatic artery (*n* ​= ​9, 10.5%), GDA (*n* ​= ​3, 3.5%), or the right hepatic artery (*n* ​= ​1, 1.2%), as shown in Table 2.

**Table 2:** Origin of right gastric artery.

Right gastric arises from	Number
Proper hepatic artery	63 (73.3%)
Common hepatic artery	10 (11.6%)
Left hepatic artery	9 (10.5%)
Gastroduodenal artery	3 (3.5%)
Right hepatic artery	1 (1.2%)

The procedure time of antegrade embolization and retrograde embolization was 4.8 ​± ​5.1 ​min (range 2–13 ​min) and 9.8 ​± ​6.5 ​min (range 5–16 ​min), respectively. The overall success rate of RGA embolization was 70.9% (61/86). In the first phase, with only antegrade embolization, the success rate of RGA embolization was 63.6% (35/55). In the second phase, with combined antegrade and retrograde embolization, the success rate was 83.9% (26/31). Retrograde embolization was successful in 12 of the 17 patients after antegrade embolization failure. The success rate of the RGA embolization was significantly higher with combined antegrade and retrograde embolization than with antegrade embolization alone (83.9% vs. 63.6%, *p* ​= ​0.047), as shown in Table 3.

**Table 3:** RGA embolization success rate and the incidence of acute gastroduodenal mucosal toxicity (AGMT) after HAIC with antegrade and retrograde embolization.

	Antegrade route	Retrograde route	Total success rate	*p* value	AGMT	*p* value
First phase	35/55 (63.6%)	0	35/55 (63.6%)		16/55 (29.1%)	
Second phase	14/31 (45.2%)	12/31 (38.7%)	26/31 (83.9%)	0.047	3/31 (9.7%)	0.037
Total	49/86 (57.0%)	12/31 (38.7%)	61/86 (70.9%)		19/86 (22.1%)	

Ectopic embolization or other complications were not observed to occur during the coil’s release.

AGMT developed after HAIC in 19 (22.1%) of the entire group of 86 patients. The incidence of AGMT in the first phase with antegrade embolization alone was 29.1% (16/55), which is significantly higher than the 9.7% (3/31) in the second phase with a combination of antegrade and retrograde embolization (*p* ​= ​0.037).

## Discussion

Gastromucosal lesions, which have been reported to occur in 3.2–47.5% of patients who have undergone repeated administration of HAIC,^5^^,^^6^^,^^15^^,^^18^ are caused by the opening of the RGA through which chemotherapeutic agents are distributed to the stomach wall. Embolization of the RGA has been proposed as a means of averting this drug-induced complication.^5^^,^^13^^,^^15^^,^^19^^,^^20^ The incidence of endoscopically confirmed mucosal lesions has been reported to be as high as 36% in patients with insufficient embolization of the RGA for HAIC via port-catheter system, while that in patients with sufficient RGA embolization at long-term follow-up was 3%.^20^ In the present study, AGMT developed after HAIC in 19 patients (22.1%), which was similar to that reported in a previous study.^20^

With regard to anatomical variations, the most commonly reported sites of divergence of the RGA are the proper hepatic artery (in 40%–52% of individuals)^12^^,^^15^^,^^21^ and the right or left hepatic artery (21%–42%).^12^^,^^15^^,^^21^ Divergence has also been reported in the common hepatic artery (in 1.5–10%)^21^^,^^22^ and the gastroduodenal artery (in 1.5–10%).^21^^,^^22^ The frequency of the sites of divergence is similar to those seen in the present study, suggesting that our subjects did not differ appreciably from other cohorts. With this, it is reasonable to assume that the RGA has a high degree of variation in its origin. Hence, identification of this vessel is imperative for regional therapy.

The RGA is usually small (less than 2 ​mm in diameter), angulated, and rich in anatomical variations. Hence, it is sometimes difficult to advance catheters selectively from the site of the hepatic artery into the RGA. Given the anastomotic arcades of the left and right gastric arteries, retrograde catheterization and embolization have proven to be effective with microcatheters via the LGA.^23^ Moreover, with the development of microcatheters that use coaxial systems, catheterization into more narrow peripheral vessels has become feasible. In this study, we succeeded in the selective catheterization of the RGA using a retrograde route from LGA in 12 of the 17 patients after failure of antegrade embolization. For the other 5 patients with redundant anastomoses, we failed to insert a microcatheter into the origin of the anastomotic branch from the LGA or were unable to advance a microcatheter into the proximal RGA. For patients with redundant anastomoses between the right and left gastric arteries, Hashimoto et al.^23^ first reported the use of intragastric gas in cases where advancing the microcatheter through the LGA toward the RGA is difficult.

This study demonstrated that the RGA embolization success rate significantly increased from 63.6% to 83.9% with the combined antegrade and retrograde embolization. Correspondingly, the incidence of AGMT after HAIC decreased from 29.1% to 9.7%.

Our study has some limitations. First, it was a retrospective review. Second, we performed retrograde catheterization of the RGA in a limited number of patients. Third, AGMT was mostly diagnosed clinically using signs and symptoms of gastroduodenal inflammation and ulceration and only 4 cases were confirmed using endoscopy. This bias is also present in the report of Inaba et al.^20^; therefore, the actual incidence of AGMT in patients undergoing HAIC may be even higher than that reported in previous studies.

In conclusion, it is very crucial to embolize the RGA as much as possible during port-catheter implantation for HAIC to reduce gastroduodenal mucosal injury. Retrograde embolization of the RGA through LGA with a microcatheter is feasible. Compared with antegrade embolization alone, the combination of antegrade and retrograde embolization can increase the RGA embolization success rate and decrease the incidence of AGMT after HAIC.

## Patient consent

Written informed consent was obtained from patients for publication of these case reports and any accompanying images.

## Ethical approval statement

All procedures performed in studies involving human participants were in accordance with the ethical standards of the institutional and/or national research committee and with the 1964 Helsinki declaration and its later amendments or comparable ethical standards. For this retrospective study formal consent is not required.

## Informed consent statement

Informed consent was obtained from all individual participants included in the study.

## Declaration of competing interest

The authors declare that they have no conflicts of interests to this work. We declare that we do not have any commercial or associative interest that represents a conflict of interest in connection with the work submitted.
